# 

**Published:** 1919-09-13

**Authors:** 


### Graduate Medical Instruction.

Through unavoidable circumstances we regret that we have to hold over until next week a monograph on this subject, probably the most important which the medical profession has to deal with efficiently and solve without further delay.

### Books for the Blind and Books for the Seeing.

THE third interim report of the Adult Education Committee, appointed by the Ministry of Reconstruction, deals with libraries and museums, and advocates a plan of co-ordination of libraries which will, it is hoped, bring about a better distribution of educational and technical books. It proposes the foundation of a central circulating library which will supplement the book collections and local libraries by supplying on loan technical books which public libraries do not provide, and works of a more specialised character than local libraries are justified in obtaining. Thus, the industrial worker, who is separated from the main centre of his industry, will be able to obtain the works most interesting to him, and the student will succeed in carrying his researches beyond the limit afforded to him by the local library. The National Institute for the Blind has opened a branch for the use of those blind persons who are in the habit of having books read to them. These books are condensed in such a way that the best passages and the vital parts are indicated to the person who reads aloud, while those portions of the work which may be described as padding or, rather, less important, are marked in such a way that they can be passed over. Most readers, whether blind or seeing, object strongly to abridged books, but there is something to be said for the plan of the "potted" book prepared for the use of the reader who wishes to cover as much ground as possible in a short time.

### Matrons' and Sisters' Appointments.

ROYAL BERKSHIRE HOSPITAL, READING.—Miss Vera Hollick and Miss Alys M. Hatton have been appointed home and night sisters. Miss Hollick was trained at the General Hospital, Cheltenham. She has been sister at the Alexandra Hospital, Queen Square, London, Salisbury Infirmary, and Royal Victoria Hospital, Boscombe. She has also done district work in Canada. Miss Hatton was trained at the Royal Salop Infirmary, Shrewsbury, where she has been holiday sister. She has been sister at the Macclesfield General Infirmary, Miller General Hospital, S.E., and ward and night sister at the General Hospital, Walsall.

GENERAL HOSPITAL, NOTTINGHAM.—Miss V. E. Jones has been appointed massage sister. She was trained at the Royal Infirmary, Bristol, and holds the I.S.T.M. certificate.

HOME HOSPITAL FOR WOMEN, STOKE NEWINGTON.—Miss Florence A. Haig Brown has been appointed matron. She was trained at St. Thomas's Hospital, where she was home sister. She has done private nursing, and also served four years under the War Office.

CLITHERO UNION, LANCASHIRE.—Miss Norah Wilson has been appointed head nurse. She was trained at St. James's Infirmary, Wandsworth, and received her midwifery training at the Dundee Maternity Hospital.

WESTHULME INFECTIOUS DISEASES HOSPITAL, OLDHAM.—Miss Mary Agnes Graham has been appointed matron. She was trained at the Beckett Street Infirmary, Leeds, and has been home sister at the Bradford Fever Hospital, and matron of the Calverley Moor Hospital, Huddersfield.

THE SANATORIUM, MIDDLESBROUGH.—Miss Ellen Macdonald has been appointed sister. She was trained at the Beckett Street Infirmary, Leeds, and the City Hospital, Seacroft, Leeds. She has also held an appointment at the Chester War Hospital, Hoole Lane.

STAMFORD, RUTLAND AND GENERAL INFIRMARY, STAMFORD.—Miss Alice Abbey has been appointed sister. She was trained at the Royal Victoria Infirmary, Newcastle-on-Tyne, where she held the post of sister and temporary sister of the skin department. She has done sister's duties under the T.F.N.S.

THE INFIRMARY, CRUMPSALL.—Miss Elizabeth Leach and Miss Elizabeth Sealey have been appointed assistant matrons. They were both trained at Crumpsall, Miss Leach later becoming night ward sister, x-ray and assistant theatre sister, and night superintendent. She holds the certificate of the Central Midwives Board. Miss Sealey has been ward sister and night superintendent, and holds the certificate of the Central Midwives Board. Miss Clara Kershaw and Miss Doris Clare have been appointed night superintendents. They were both trained at Crumpsall, later becoming ward sisters, and both hold the certificate of the Central Midwives Board.

VICTORIA HOSPITAL, BLACKPOOL.—Miss Nora Martin has been appointed matron. She was trained at the Derbyshire Royal Infirmary, where she has been assistant matron. She has also been sister-in-charge at the Station Road Auxiliary Hospital, Blackpool.

EYE INFIRMARY, WOLVERHAMPTON.—Miss Minnie Williams has been appointed sister. She was trained at the Bolingbroke Hospital, London, and at the Wolverhampton and Midland Counties Eye Infirmary, where she has been staff nurse.

WASSEL GROVE CONVALESCENT HOME, STOURBRIDGE.—Miss Olive Langhorn, A.R.R.C., has been appointed matron. She was trained at the Royal Infirmary, Manchester, where she was temporary sister. She has held the appointment of sister at Horton War Hospital, Epsom, and matron of Harborne Hall Auxiliary Hospital, Birmingham.

CHILDREN'S HOSPITAL, LOWER SYDENHAM.—Miss Ethel Butcher has been appointed home sister. She was trained at the Woolwich Infirmary, Plumstead, and has been ward sister, acting night sister, and home sister at the Holborn Infirmary, Highgate, and sister at the Mile End Military Hospital. She has done private nursing, and holds the certificate of the Central Midwives Board.

TANKERTON HOSPITAL, WHITSTABLE.—Miss Dorothy Metygar has been appointed theatre sister. She was trained at St. Mary's Hospital, Paddington, and has been theatre sister at the Samaritan Hospital, Marylebone Road, London.

SWANSEA GENERAL AND EYE HOSPITAL.—Miss Kathleen M. Smith has been appointed sister. She was trained at the Royal Halifax Infirmary and has been staff nurse at the City of Leeds Fever Hospital, sister at the Hospital for Women, Derby, and the General Hospital, Cheltenham, night superintendent County Hospital, York, and sister, assistant matron, and matron in the T.F.N.S.

ST. JAMES'S INFIRMARY, BALHAM, S.W.—Miss Laura Brown has been appointed assistant matron. She was trained at the Royal Devon and Exeter Hospital, where she was on the private staff, and at the Hospital for Women, Soho. She has served at the South-Western Fever Hospital, Stockwell, as assistant matron of the Royal Portsmouth Hospital, and as Naval Reserve Sister at Plymouth and Haslar.

### Presentation.

A VERY interesting ceremony took place in the London Temperance Hospital on Friday, the 29th ultimo, when Dr. Catherine M. Ironside was presented by the Board of Management with a resolution of thanks and a clock as an appreciation of the very capable and humane way in which she carried out her duties as resident medical officer for over two and a half years. Sir Vezey Strong, who presided, spoke in glowing terms of these services.

